# Assessing the Consistency and Microbiological Effectiveness of Household Water Treatment Practices by Urban and Rural Populations Claiming to Treat Their Water at Home: A Case Study in Peru

**DOI:** 10.1371/journal.pone.0114997

**Published:** 2014-12-18

**Authors:** Ghislaine Rosa, Maria L. Huaylinos, Ana Gil, Claudio Lanata, Thomas Clasen

**Affiliations:** 1 Faculty of Infectious and Tropical Diseases, London School of Hygiene & Tropical Medicine, London, United Kingdom; 2 Instituto de Investigación Nutricional, Lima, Peru; 3 Department of Environmental Health, Rollins School of Public Health, Emory University, Atlanta, Georgia, United States of America; U. S. Salinity Lab, United States of America

## Abstract

**Background:**

Household water treatment (HWT) can improve drinking water quality and prevent disease if used correctly and consistently by vulnerable populations. Over 1.1 billion people report treating their water prior to drinking it. These estimates, however, are based on responses to household surveys that may exaggerate the consistency and microbiological performance of the practice—key factors for reducing pathogen exposure and achieving health benefits. The objective of this study was to examine how HWT practices are actually performed by households identified as HWT users, according to international monitoring standards.

**Methods and Findings:**

We conducted a 6-month case study in urban (n = 117 households) and rural (n = 115 households) Peru, a country in which 82.8% of households report treating their water at home. We used direct observation, in-depth interviews, surveys, spot-checks, and water sampling to assess water treatment practices among households that claimed to treat their drinking water at home. While consistency of reported practices was high in both urban (94.8%) and rural (85.3%) settings, availability of treated water (based on self-report) at time of collection was low, with 67.1% and 23.0% of urban and rural households having treated water at all three sampling visits. Self-reported consumption of untreated water in the home among adults and children <5 was common and this was corroborated during home observations. Drinking water of self-reported users was significantly better than source water in the urban setting and negligible but significantly better in the rural setting. However, only 46.3% and 31.6% of households had drinking water <1 CFU/100 mL at all follow-up visits.

**Conclusions:**

Our results raise questions about the usefulness of current international monitoring of HWT practices and their usefulness as a proxy indicator for drinking water quality. The lack of consistency and sub-optimal microbiological effectiveness also raises questions about the potential of HWT to prevent waterborne diseases.

## Introduction

Unsafe drinking water is a major cause of diarrheal death and disease, especially for young children and vulnerable populations in low-income countries. In 2012, unsafe drinking-water, together with poor sanitation and hygiene, accounted for an estimated 842 000 diarrhoeal deaths, which equates to 1.5% of the total burden of disease and 58% of diarrhoeal diseases [1].

Household water treatment (HWT), including boiling, chlorination, filtration and solar disinfection, can improve water quality at the point of use if combined with safe storage to prevent post-collection contamination. Systematic reviews of water quality interventions suggest that HWT is effective at improving drinking-water quality and in preventing diarrhoea [2]–[4]. Although there is evidence that the health impact from HWT may be exaggerated due to reporting bias, the WHO and UNICEF have recommended the use of HWT for populations relying on unsafe supplies as part of a comprehensive strategy to prevent diarrhoea [5].

The WHO/UNICEF Joint Monitoring Programme for Water Supply and Sanitation (JMP) is charged with monitoring water and sanitation practices and progress towards international goals. In 2006, the JMP recommended that two questions be added to nationally-representative household surveys to gather information on whether and how HWT is practiced. The intent was to obtain baseline prevalence data on HWT and to explore whether responses to the questions could serve as an indication of water quality in the home [6], [7]. The results from 67 low- and middle-income countries suggest that more than 1.1 billion people report treating their water prior to drinking [8]; additional data from China brings the overall figure to at least 1.8 billion [9].

While the household-based surveys used by the JMP may be a practical means of collecting data on HWT practices at the regional and national level, there are questions about the reliability and usefulness of these data for public health purposes [10], [11]. First, since study participants tend to over-report “good” practices [12]–[15], it is likely that they exaggerate HWT practices [16]–[19]. Second, little is known about the manner in which HWT is practiced among these populations of self-reported users, especially regarding its level of compliance and its effectiveness—key factors for reducing exposure and achieving the health benefits associated with HWT [20]–[22]. Field studies have reported that HWT does not remove the full risk to waterborne pathogens [23]–[26]. Third, even householders who treat their water at home may continue to be exposed to untreated water due to (i) failing to drink treated water exclusively when at home, (ii) drinking untreated water when away from home, and (iii) not treating and storing their water effectively [27]–[29].

With funding and support from the JMP, case studies were undertaken in India, Zambia and Peru to investigate these issues, to document HWT practices among self-reporting HWT-users, to explore the extent to which existing JMP surveys capture key aspects of these HWT practices, and to assess the implications of these practices on disease prevention. Here we report results from Peru, an upper middle-income country where according to JMP figures, 82.8% of households report treating their water prior to drinking, the vast majority (77.6% of households) by boiling [30].

## Methods

### Study design

**Figure 1:**
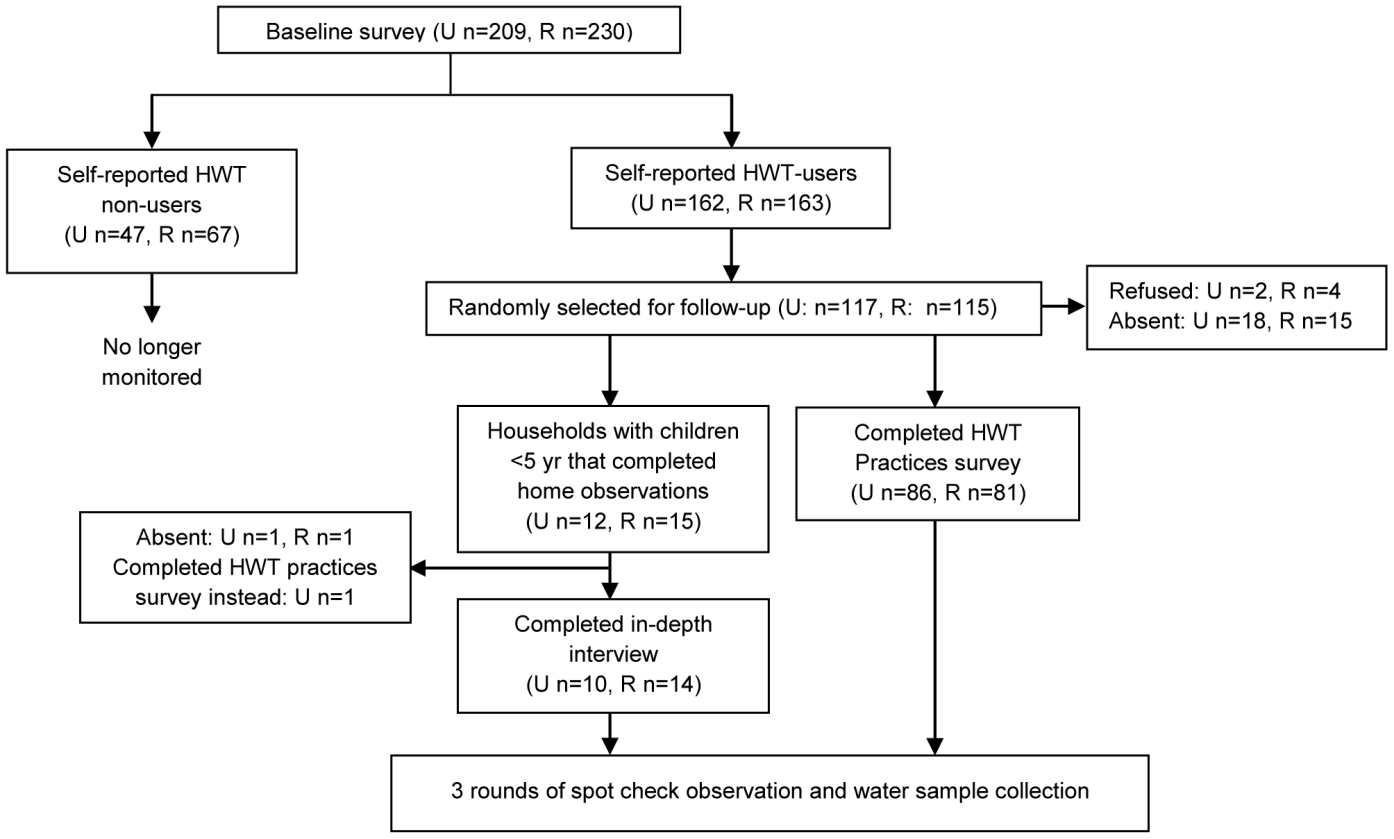
Schematic representation of the study design.

The study followed a prospective panel design, in which a selection of households that claimed to practice HWT was followed for 6–7 weeks. After receiving complete details regarding the study and obtaining consent from eligible participants, a baseline survey was undertaken to collect information on demographics, socio-economic characteristics, water handling, HWT and sanitation practices as well as fuel and cooking practices. Our baseline survey followed closely the surveys used by the Demographic and Health Surveys (DHS) and the Multiple Indicator Cluster Surveys (MICS), which the JMP heavily relies on for monitoring access to water and sanitation [6]. Data collection tools were translated into Spanish and piloted before use. A random selection of 115 households from those households identified as HWT practitioners during the baseline survey (i.e. responded affirmatively to the JMP core question on HWT —“Do you treat your water in any way to make it safer to drink?”) were followed-up. As detailed further below and in Fig. 1, follow-up consisted of (A) either (i) a second household HWT practice survey, or (ii) structured observations in the home together with in-depth interviews (IDI), plus (B) spot-check observations and water sampling. The study was independently conducted in both urban and rural settings.

### Study setting, participant eligibility

The study was conducted from November 2011 through March 2012 in the northern highlands of Peru, in the Province of San Marcos, Cajamarca. The urban setting was in San Marcos, the capital city of the province; the rural setting was in three neighbouring villages, Illuca, La Laguna and La Manzanilla, 70 km north of San Marcos city. The urban community consists mainly of retail, services and the commercial sector with most people living in cement buildings, while the rural communities comprise mostly of subsistence farmers living in houses made out of earthen floors and adobe walls. In the rural setting, all households in the three villages were approached for participation. In the urban setting, due to the unavailability of a sampling frame, the city was subdivided into seven sectors of approximately similar size and systematic sampling was used to select the participating households. In both settings, study participants were recruited through door-to-door visits. Households were eligible to participate if (i) at least one household member lived permanently in the household, (ii) in those cases where only one member resided in the household, that the household member had no impediments to providing informed consent, and (iii) the household was not planning on moving in the next three months.

### Sample size

The sample size calculation was based on precision estimates rather than power estimates as we were not interested in comparing groups but rather in determining, with a given precision, certain characteristics of reported HWT-users. Aiming for a precision of ±10% and an anticipated proportion of interest of 0.7, a minimum of 80 households reporting HWT-use was required. This was increased to 115 households to account for potential loss to follow-up. Using the ENDES 2004–6 [31] figures on HWT-use in urban and rural settings for the province of Cajamarca (75.7% and 53.7%, respectively) it was estimated that 152 and 217 households would need to be recruited in urban and rural settings, respectively to identify the required number of households claiming to perform HWT.

### Home observations and in-depth interview

Structured observations were undertaken to assess whether, and how often, HWT was performed and to determine whether treated water was consumed exclusively in the household or whether it was supplemented with untreated water from other sources. Approximately 15% of the participating households were purposely selected to complete three days of direct observations in their home followed by an in-depth interview administered at the end of the observation period. Only households with at least one child <5 years of age were invited to participate in the home observations. Observation times were pre-arranged with the female head of the household and were scheduled to last between 4–6 h. Observations were presented to have broader public health aims to minimize drawing attention to water handling or HWT practices. Female enumerators undertook all observations.

### Reported HWT practices survey

The remaining 85% of the participating households completed a second survey (hereinafter, the “HWT practices survey”). In this survey, participants were questioned a second time about their HWT practices, allowing the assessment of the concordance of the HWT-use responses with those obtained during baseline. The second aim of the survey was to obtain further insight into HWT practices, with special focus on adherence to the practice: (i) frequency of use, (ii) consumption of treated water in the home, and (iii) exclusive drinking of treated water (non-supplementation) by the survey respondent and children under 5 years of age living in the household.

### Observational spot-check visits

All participating households were visited on two further occasions, at intervals of four to twelve days to complete an observational spot-check visit. The main aims of these spot-check visits were to determine whether the water available for drinking was reported to have been treated and if so, whether the necessary materials to perform HWT were available (an objective proxy measure of HWT-use). Data were also gathered on other potential objective proxy measures of HWT-use, such as water in the storage container of water filters for those reporting filtration, free chlorine residuals (FCR) in the water for those reporting chlorination, and temperature of water for those reporting boiling. These visits were unannounced. To reduce the potential of reactivity in future visits, spot-check observations of the sanitation and hand-washing facilities were undertaken at the start of each visit.

### Water quality

To characterise the microbiological quality of the drinking-water of those households identified at baseline to report following a HWT method deemed “adequate” by the JMP (i.e. reported boiling, filtration, chlorination or solar disinfection [6]), we collected one sample of drinking-water at unannounced visits from each participating household during the HWT practices survey (or IDI) and at the two observational spot-check visits. Fieldworkers were instructed to ask the participant: “If you or someone in this household wanted a drink of water right now, what water would you drink from?” In the case that there were children <5 years in the household, fieldworkers were instructed to ask in reference to the children <5 years. Efforts were made to identify all possible containers of stored drinking-water. A 125 mL sample was collected aseptically from the most frequently used container. Furthermore, to evaluate the effectiveness of the reported HWT method, immediately thereafter, a paired “source water” sample was collected from the source (standpipe, well, etc.) from which the household reported drawing the drinking-water. All water samples were collected in sterile Whirl-Pak bags (Nasco, Fort Atkinson, WI) containing a tablet of sodium thiosulphate to neutralise any halogen disinfectant. Samples were placed on ice and processed within 4 h of collection to assess levels of thermotolerant coliforms (TTC), an indicator of faecal contamination [32]. Microbiological assessment was performed using the membrane filtration technique [33] on membrane lauryl sulphate medium (Oxoid Limited, Basingstoke, Hampshire, UK) using a DelAgua field incubator (Robens Institute, University of Surrey, Guilford, Surrey, UK).

### Data analysis

All data were double entered into Microsoft Excel 2008 and analysed using STATA version 10 (Stata Corp., College Station, TX). Due to over-dispersion of the water quality data and excess 0 values, the arithmetic as well as the Williams means are presented. The Williams mean is calculated by adding 1 to all the data values, then taking the geometric mean, then subtracting 1 again [34]. To assess whether the drinking-water of reported adequate HWT-users identified at baseline was of higher quality than the source water, the log differences of the paired source-drinking water samples was calculated for each of the three individual rounds of follow-up. Overall, source and drinking-water TTC counts were compared using random effects negative binomial regression to account for (i) repeated observations within households, and (ii) the positive skewed distribution of the TTC counts. For the purpose of analysis, plates that yielded coliform forming units (CFUs) that were too numerous to count (TNTC) were assigned a value of 500 CFU. For the home observations, simple descriptive analysis, involving counting of relevant events (such a water drinking events or use of HWT) and descriptions of HWT events was undertaken. We measured a household's consistency of reporting HWT as the number of times a households reported to perform HWT (at baseline and during the HWT practices survey or IDI) and the number of times they reported to have treated water at the three water collection visits (HWT practices survey or IDI and observational spot-check visits). We further assessed consistency of use by crosschecking reported daily use or non-supplementation and the availability of treated water (based on self-report) at the time of the interview.

### Ethics

This study was reviewed and approved by the Ethics Committee of the London School and Hygiene and Tropical Medicine (Reference No. 5696 dated 13 April 2010) and by the internal Research Ethics Committee of the Instituto de Investigación Nutricional (Reference No. 319-2011/CEI-IIN dated 8 August 2011). Informed written consent to participate in the research was obtained from the head of the household or spouse of each participating household.

## Results

### Baseline characteristics

A total of 209 and 230 households were enrolled and completed the baseline survey in the urban and rural studies, respectively (S1 Table). The urban study population consisted of 807 individuals (mean 3.9 occupants per household), of whom 12.4% were children under 5 years. 49.3% of heads of households had secondary or higher education. Most households had access to piped water in their dwelling or plot (96.6%) and over three quarters of the urban study population relied on pour or pour flush toilets connected to the sewer system. Gas was the most common cooking fuel in the area (58.4%), followed by firewood (39.7%).

The rural study population consisted of 813 individuals (mean 3.5 occupants per household), of whom 10.3% were children under 5 years. Only 17.4% of heads of households completed secondary or higher education. As in the urban community, most households relied on piped water into their dwelling or yard (85.6%). However, in this setting most households (74.3%) relied on pit latrines with slabs. Firewood was by far the most common cooking fuel (96.5%).

Most households in both settings (urban (U): 77.5%, rural (R): 70.9%) reported treating their water prior to drinking. In both settings, boiling was the most common method of treatment (U: 77.5%, R: 69.6%).

The refusal rate to complete the baseline survey was 28.0% and 8.6% in the urban and rural settings, respectively.

### HWT practices survey

A total of 86 and 81 households completed the HWT practices survey in the urban and rural communities, respectively (Table 1). Consistent with the baseline survey, the majority of households in both settings relied on piped water for drinking purposes. There was a strong sense of mistrust towards the drinking-water supply, with 84.9% of urban households and 50.6% or rural households believing that their source was not safe for human consumption. Both urban (89.5%) and rural (79.0%) households reported storing drinking-water at home. Narrow-mouth containers that may be more protective against contamination were as common as wide-mouth containers in the urban setting, but wide-mouth containers were much more prevalent in the rural setting.

**Table 1:** Summary of reported HWT practices as reported during the HWT practices survey.

Characteristic	Urban		Rural	
	n	%	n	%
Number of households	86		81	
Water handling practices				
Store drinking water at home	77	89.5	64	79.0
Percentage of households with>1 type of storage container	23	29.9	11	17.2
Percentage of households with a wide opening container^1^	47	61.1	56	87.5
Percentage of households with a narrow opening container^1^	47	61.1	18	28.1
Report covering drinking container	74	98.7	62	96.9
**Access drinking water (only for wide vessels)**				
Dip a glass	21	44.7	42	75.0
Use a ladle	18	38.3	3	5.4
Use a tap	3	6.4	3	5.4
Other	5	10.6	2	3.6
**HWT practices**				
Reported HWT-use	81	94.2	68	84.0
Reported method				
Boil only	75	92.6	66	97.1
Boil & use chlorine or bleach	5	6.2	2	2.9
Boil & let stand and settle	1	1.2	0	0.0
All household members consume the treated water	75	92.6	61	89.7
Use treated water for other purposes	13	16.1	25	36.8
HWT performed year around	76	93.8	55	80.9
Reported frequency of HWT-use				
Daily	64	79.0	43	63.2
Every 2–4 days	15	18.5	22	32.4
Every 8–15 days	0	0.0	2	2.9
Rarely	2	2.4	1	1.5
Cross-checking reported data on daily HWT-use				
Last treatment performed>2 days prior to survey (among reported daily users)	2	3.1	4	9.3
Availability of treated water if reported daily use^2^	57	90.5	25	62.5

1 Respondents may report multiple types of container, so the sum of containers may exceed 100 percent.

2 Among those with available water at the time of the visit (U: n = 63, R: n = 40). Based on self-report.

Upon probing with the JMP HWT question a second time during this survey, 94.2% and 84.0% of households reported HWT-use in the urban and rural setting, respectively. Boiling was the predominant method in both settings. The majority of households (U: 93.8%, R: 80.9%) reported treating their drinking-water throughout the year, with the others only undertaking treatment at times of illness or during the wet season. A large proportion of households (U: 85.2%, R: 89.7%) reported practicing treating their water for years, with an average of 11.0 and 8.2 years in the urban and rural setting, respectively. Almost all participating households (U: 92.6%, R: 89.7%) reported that all family members would consume the drinking-water equally. Females were largely in charge of performing HWT.

Over three quarters of the participating urban households reported practising HWT on a daily basis; daily use was slightly lower in the rural context (63.2%). However, crosschecking this data showed some discrepancies: while 90.5% (95% CI: 83.0–97.9, n = 63) of urban households had treated water at home at the time of sampling, the figure was only 62.5% (95% CI: 46.8–78.2, n = 40) for rural households. Three-quarters of urban households reported that they would treat more drinking-water the same day if their supply was exhausted; 5.1% reported that there would be no need to treat a second time while the remaining households (20.3%, 95% CI: 12.7–30.8, n = 79) would wait until the next day and consume untreated water until then. In the rural setting, 52.9% of households would treat more water the same day, 4.4% reported that there would be no need while 42.7% (95% CI: 31.2–54.9, n = 68) would wait until the next day.

Reported supplementation of treated water by consuming untreated water was common among the urban and rural populations, with 34.6% (95% CI: 23.9–45.1, n = 81) and 67.7% (95% CI: 56.2–79.1, n = 68) of respondents acknowledging supplementation, respectively (S2 Table); 17.9% and 23.9% reported doing so on a daily basis. However, there was evidence of over reporting of non-supplementation, especially in the rural setting, with 5.9% (95% CI: 0.0–12.5, n = 51) and 21.1% (95% CI: 0.9–41.2, n = 19) of those who claimed not to supplement treated water having stored untreated drinking-water at the time of the visit. Consumption of untreated water among children under 5 years was almost as common, with 27.6% (95% CI: 28.4–44.8, n = 29) and 55.0% (95% CI: 31.1–78.9, n = 20) of caretakers in urban and rural settings reporting that their children consumed untreated water when at home.

### Home observations

A total of 12 and 15 households were observed for a mean of 12.8 and 14.1 h in the urban and rural settings, respectively (S3 Table). Among these, 8 urban and 16 rural HWT events were observed across the entire sample and duration of the observations. Only nine urban (81.2%) and six rural (46.2%) households reported performing HWT on a daily basis in the complementary IDI that followed the home observation. During the three days of observation among these households that reported daily HWT, 5 of the 9 urban households and 3 of the 6 rural households were not observed to actually treat their water at all while most other did so only once.

A total of 66 and 82 water drinking events were observed in the urban and rural setting. No drinking events were observed in 1 urban and 2 rural households during the entire observation period. Though fieldworkers were instructed to focus on drinking-water vessels and drinking events, in several instances it was difficult to determine the HWT status of the drinking-water (i.e. vessels that already had water prior to the arrival of the fieldworker), and so a large proportion of the water drinking events in the urban setting (42.4%) and a few in the rural setting (2.6%) were classified as “unknown“. Of those event that could be classified, however, 46.6% of the urban events and 65.6% of the rural events involved consumption of untreated water. In many cases mothers saw and permitted their children to drink untreated water, frequently directly from the yard tap.

### In-depth interviews

Overall, 10 and 14 households completed the IDI in the urban and rural setting, respectively. During the in-depth interview that followed the observations, all but one household in the rural context reported treating their water, in all cases by boiling, though two urban households reported occasionally using chlorine too. The majority of urban households reported that their water was not safe for consumption (80%), while only a minority of the rural participants believed so (14.3%) or were uncertain (14.3%). All households reported practising HWT for health reasons. In all rural cases and in half of urban cases, the respondent made specific reference to microbes in the water. However, a third of urban and rural respondents believed that boiling water could prevent coughs and chest-related illnesses as well as diarrhoeal diseases. Sixty percent of urban respondents and 90.3% of rural respondents reported that they had heard of HWT from their local health centre, although other sources of information such as family, tradition, and government-run child-caring services were also mentioned.

All but one urban respondent and half of rural participants reported boiling their water on a daily basis. Householders in both settings reported treating their water throughout the year; however, a minority of participants acknowledged that they would practice HWT more seriously during the months of heavy rain (U: 10%, R: 7.7%).

All respondents reported that all family members including the children and the elderly would consume the treated water. However, some respondents acknowledged that certain family members would drink less treated water (and consume more untreated water) (U: 20.0%, R: 15.4%). Seventy percent of participants in both settings reported consuming untreated water at home. Regarding under 5s, 45.5% of urban and 81.8% of rural participants reported that their children consumed untreated water when at home.

Further details of boiling specific issues gathered during the in-depth interviews are presented as supporting information (S1 Text).

### Consistency analysis

A total of 94.8% and 85.3% of urban and rural households respectively, responded affirmatively to the HWT-use question in both occasions (Table 2). Among those that reported affirmatively in both occasions, over 90% of households in both settings were consistent in their reported method of HWT.

**Table 2:** Consistency of HWT-use among households that self-reported performing HWT at baseline in the urban and rural communities.

Characteristic	Urban			Rural		
	n	%	95% CI	n	%	95% CI
Consistent reporting of HWT-use in the baseline and HWT practices survey/IDI^1^	91	94.8	87.9–97.9	81	85.3	76.5–91.2
Consistent reporting of HWT method among those reporting use in both occasions	82	90.1	81.9–94.84	74	91.4	82.7–95.9
Consistent reporting in all five HWT reporting events^2^	51	67.1	55.6–76.9	14	23.0	13.9–35.5
Number of home visits with available treated water (based on self-report) 2						
Three	51	67.1	55.6–76.9	14	23.0	13.9–35.5
Two	13	17.1	10.1–27.5	13	21.3	12.6–33.7
One	4	5.3	1.9–13.5	13	21.3	12.6–33.7
None	8	10.5	5.3–19.9	21	34.4	23.4–47.5
*Sub-group analysis*						
Claimed to have treated water at all three collection points:						
Reported daily HWT-use^3^	43	76.8	63.6–86.3	9	28.1	14.8–46.9
Reported non supplementers^4^	34	81.0	65.6–90.5	6	42.9	18.3–71.6
Reported supplememters^5^	17	58.6	39.2–75.7	8	21.1	10.5–37.6

1 Among households that completed both visits (U: n = 96, R: n = 95).

2 Among households that completed all five visits and had water available at all three points (U: n = 76, R: n = 61).

3 Among households that had water at all three points and reported daily HWT-use (U: n = 56, R: n = 32).

4 Among households that had water at all three points and reported to be non-supplementers (U: n = 42, R: n = 14).

5 Among households that had water at all three points and reported to be supplementers (U: n = 29, R: n = 38).

Of the households being followed-up, 76 (78.4%) and 61 (63.5%) completed all three rounds of follow-up and had drinking-water available at all three unannounced visits in the urban and rural setting, respectively. In the urban setting, two thirds of households (95% CI: 55.6–76.9, n = 76) were consistent with their reported HWT practices. These households reported HWT-use in both interviews and claimed to have treated drinking-water in all three water collection visits. On the other hand, 17.1% of households claimed only twice to have treated water at the time of water collection, and 10.5% of households (95% CI: 5.3–19.9, n = 76) had untreated water on all three occasions. In the rural setting, inconsistent use was more prevalent, with only 23.0% of households (13.9–35.5, n = 61) reporting HWT-use at both instances and claiming to have treated water at all three collection points. Furthermore, just over a third of households (95% CI: 23.4–47.5, n = 61) had no treated water at all three collection visits.

Among reported daily users, 72 urban households reported daily use, of which 56 (77.8%) had drinking-water at all three collection points, 43 of which (76.8%) reported to have treated water at all three instances. In the rural counterpart, 48 households reported daily HWT, of which 32 (66.7%) had drinking-water at all three water collection visits; only 9 of these (28.1%) reportedly had treated water at all three visits.

Inconsistencies in HWT-use among those households that reported not consuming untreated water were also observed (Table 2). Although reported non-supplementers were more likely to have treated water on all three collection points than supplementers (U: 81.0% *vs*. 58.6%, n = 71, *p* = 0.04; R: 42.9% *vs*. 21.1%, n = 52, *p* = 0.12), a sizable proportion did not have treated water at all three visits.

### Water quality

**Figure 2:**
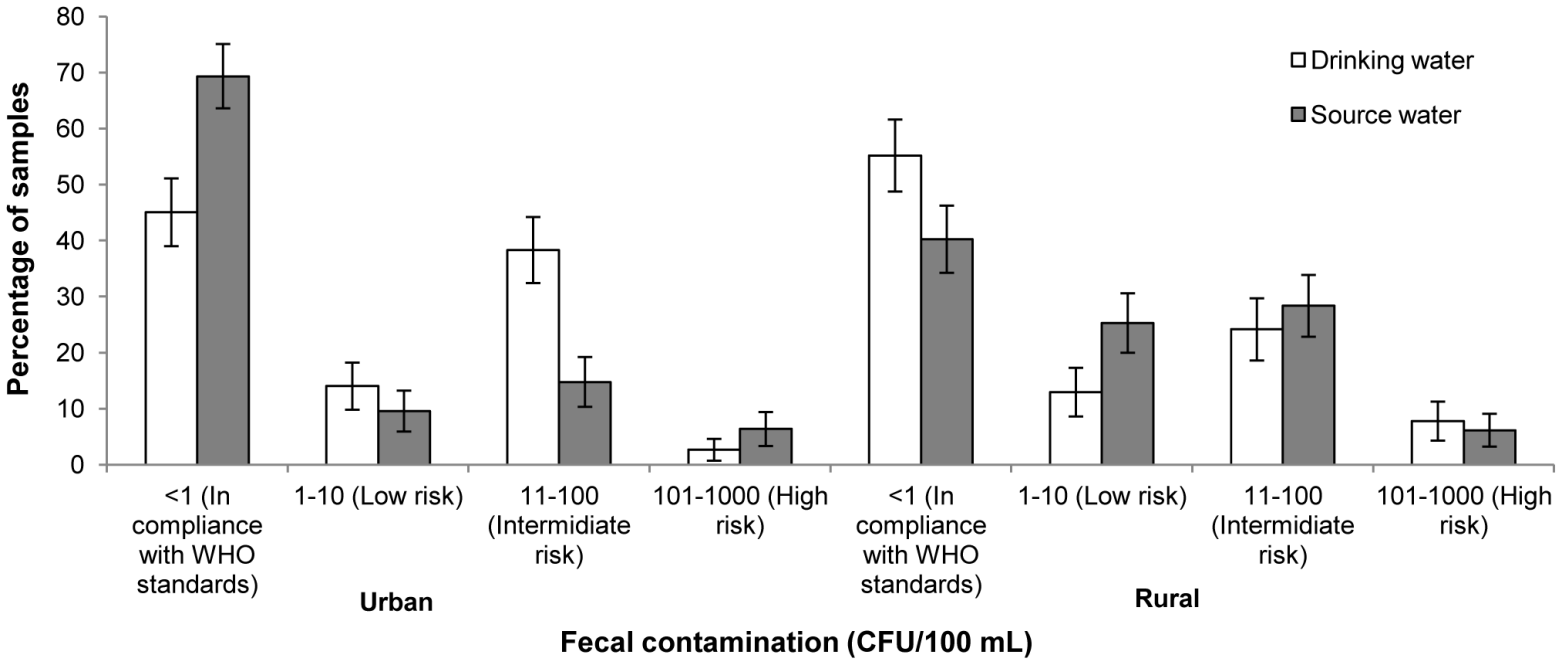
Faecal contamination in water samples of households claiming to use adequate methods of HWT at baseline.

Among the urban population of self-reported practitioners of adequate HWTS that were followed-up (n = 97), drinking-water sampled in the home had less faecal contamination than source water. Overall, 69.3% (95% CI: 63.3%–75.1%) of drinking-water samples were free of TTC compared to 45.1% (95% CI: 39.0%–51.1%) of source samples (Fig. 2). Paired water samples showed a mean log_10_ reduction of 0.14-0.44 during the different rounds of follow-up (Table 3). For a distribution of the mean log_10_ reduction refer to S1 Figure. Overall, drinking-water had 57% lower mean TTC counts than source water (*p*<0.001). Drinking-water quality in the rural context showed a smaller yet significant improvement over source water. Overall, 55.2% (95% CI: 48.7–61.6) of drinking-water samples were free of TTC compared to 40.2% (95% CI: 34.2–46.2) of source samples. Mean log_10_ reduction of 0.06–0.09 were observed in the three rounds of follow-up, with drinking-water showing overall 29% lower TTC counts than source water (*p* = 0.01). Nevertheless, only 46.3% of urban and 31.6% of rural households had drinking-water free of TTC on all completed follow-up visits.

**Table 3:** Summary statistics of the microbiological quality of samples of source and drinking water collected at each follow-up visit.

	Source					Drinking					Paired samples					
	n	AM	95% CI	WM	95% CI	n	AM	95% CI	WM	95% CI	n	Mean Log_10_ difference	95% CI	% with>1 Log_10_ reduction	n	% with>1 Log_10_ reduction among sources TTC>0
**Rural**																
Visit 1	92	22.9	9.7–36.1	3.7	3.3–6.6	87	55.2	17.0–93.5	2.8	1.5–4.9	84	0.06	−0.2–0.3	13.1	46	23.9
Visit 2	90	20.0	7.7–32.3	4.0	2.6–6.1	79	23.0	7.7–38.4	3.3	1.9–5.4	79	0.09	−0.1–0.3	12.7	47	21.3
Visit 3	79	27.9	16.8–39.1	5.9	3.6–9.3	66	39.7	7.5–71.8	4.6	2.5–7.9	66	0.07	−0.1–0.3	13.6	42	21.4
**Urban**																
Visit 1	92	19.7	13.6–25.9	3.7	2.2–5.9	89	41.2	11.2–71.2	2.1	2.1–3.7	85	0.14	−0.1–0.4	21.2	37	48.7
Visit 2	86	31.0	19.4–42.6	7.6	4.7–11.8	82	37.3	10.9–63.6	2.0	1.0–3.5	78	0.44	0.2–0.7	34.6	46	58.7
Visit 3	86	24.0	10.8–37.3	4.4	2.7–6.8	80	46.0	6.3–85.8	2.4	0.6–2.5	76	0.36	0.1–0.6	25.0	44	43.2

AM =  Arithmetic mean, WM =  Williams mean.

Moreover, a sizable proportion of the drinking-water among the participants that self-reported HWT at baseline was not treated at the time of sample collection, especially in the rural context. Overall, 81.6% and 47.1% of drinking-water samples collected in the urban and rural studies were reportedly treated. Analysis of samples according to self-reported HWT status at time of collection showed that reportedly treated water samples were of significantly higher quality than source water in both settings. Treated drinking-water had 67% and 58% lower mean TTC counts than source water in the urban and rural context (*p*<0.001 in both settings). Untreated water was of no better quality than source water. Overall, 76.9% and 69.5% of reportedly treated water was less faecally contaminated than source water to <1 CFU/100 mL or maintained the quality of safe sources, as opposed to 40.0% and 41.5% of untreated drinking-water samples.

Overall 2.5% of samples from urban households and 1.2% of samples from rural households yielded plates that were TNTC.

## Discussion

This study presents a snapshot of the HWT practices in an urban and a rural community in northern Peru of households that claimed to practice HWT using the monitoring approach employed by the JMP. In our study communities, 77.5% and 70.9% of the participating households responded affirmatively to the JMP question on HWT-use, proportions that are similar to the current JMP figures for HWT-use in the urban setting (75.7%) but somewhat higher for the rural setting (53.7%). Consistent with the JMP data, boiling was the most prevalent method for water treatment in both settings.

While more than 85% of urban and rural households in our study population report in surveys that they treat their water prior to drinking it, and claim to follow positive HWT-related practices (daily use, consumption of treated water by all household members and reporting water treatment through the year), follow-up showed these reports to exaggerate actual practices. A sizable proportion of participants, including young children, acknowledged also consuming untreated water when at home, especially in the rural context. This is consistent with previous research in Peru that found it common for mothers who had reported treating their water to allow their children to consume untreated water [35]. This was also observed during the home observations in both settings.

Furthermore, a substantial number of households, especially in the rural context, relied on untreated water during the follow-up period. This lack of treated water at collection visits has been reported in other studies of actual HWT practices. In rural Cambodia, despite>90% of households reporting that they use boiled water, on average, only 31% of water samples collected were boiled [29]; while in peri-urban Zambia, 40% of water samples from self-reported boilers were untreated [28].

Our results suggest that household surveys of self-reported HWT-use fail to account for inconsistent compliance and for consumption of untreated water. Given that recent research highlights the importance of high compliance to achieve the protective effects associated with HWT [20], current estimates of HWT may be misleading if used to estimate the potential contribution of HWT in providing protection against waterborne diseases.

Additionally, the study provided suggestive evidence of reporting bias of certain HWT-related practices such as non-supplementation with untreated water and frequency of HWT-use. This might hamper efforts to evaluate and monitor the consistency of HWT-use. This data also highlights the need for better indicators to assess consistent use and the need to develop and implement, in a more rigorous manner, tools for monitoring compliance in HWT field studies and HWT dissemination programs.

These results also suggest that the self-reported HWT estimates may be more misleading in rural settings, which may be at higher risk of waterborne infections due to lower access to safe water, sanitation and to health care. In this study, rural households who claim to practice HWT were more likely to (i) report drinking untreated water at home, (ii) over-report daily use or supplementation, and (iii) have untreated drinking-water at collection points.

Furthermore, data from the in-depth interviews indicate that lack of knowledge is not a reason for not practicing HWT. Most of the participants interviewed were well aware of the reasons for boiling their water; however this did not stop them from consuming untreated water at times. This disconnects between knowledge and action has been shown in many other environmental health interventions [12], [36].

Water quality results reported here are generally consistent with recent studies of the effectiveness of boiling in reducing thermotolerant coliforms, which have found that although boiling significantly improves the microbiological quality of water, it does not fully remove the potential risk of waterborne pathogens [23]–[25], [28], [29]. In this study, participants that responded affirmatively to the JMP core question on HWT-use had water of significantly higher quality than their source water, though the difference was almost negligible in the rural context. However, less than half of households had water that was free of TTC at all three water collection visits, probably reflecting sub-optimal compliance in these communities. When we took into account the self-reported HWT status of the drinking-water, treatment appeared to significantly improve the microbiological quality of the drinking-water in both settings. Moreover, a proportion of samples still remained contaminated, potentially indicating re-contamination in the home. It should be noted that in both urban and rural communities the quality of the source water was much higher than in many other published studies in low- and middle-income countries, potentially limiting the chance of detecting an improvement in quality associated with HWT-use and limiting the size of the mean log_10_ reductions observed in the communities.

There are important limitations inherent in this study that impact the internal and external validity of the results. First, the study communities were not randomly selected and may not be representative of the country as a whole, much less other countries and settings. Second, the refusal rate to complete the baseline survey in the urban setting was relatively high (28%); this could have implications for the representativeness of our sample. Third, we cannot rule out the potential for reactivity due to repeated follow-up visits [37]. Fourth, the study was conducted over a relatively short period of time during the Andean rainy season; it is possible that the use of HWT and compliance differ by season. Though in the study we questioned about the seasonal use of HWT and most participants responded that HWT would be performed all year round, it is possible that (i) this is subject to courtesy bias, and (ii) HWT is performed but that adherence may be lower (i.e. possibly due to a reduced perceived risk). Fifth, due to logistical difficulties in undertaking home observations, findings or associations made from these were based on a limited number of observation points. Therefore, most of the evidence from this approach should be taken as suggestive evidence only. Furthermore, due to the restrictions in the number of hours that we could observe the participants, it is possible that we missed HWT events, thus restricting the potential to classify households as users or non-users. Additionally, the differences observed in reported HWT could have been due to differences in HWT-use over time. However, this would be a finding on its own, as it would show that the JMP core question on HWT-use is sensitive to when the question is asked, thus showing poor repeatability. Finally, a major limitation of this research is the extensive reliance on self-reported data to assess actual HWT practices and compliance. This might be of particular importance in determining the microbiological effectiveness of HWT as the HWT-status of the drinking-water was based on self-report. Thus the actual effectiveness of HWT could have been miscalculated if a proportion of the drinking- water identified as treated had been misclassified due to self-report.

Subject to these limitations, these results raise questions about the usefulness of the current approach of assessing HWT practices using the JMP core questions. An affirmative response to HWT questions in a single cross-sectional survey may provide little indication of drinking-water quality in the home or actual use of HWT. The JMP is moving to actual assessments of drinking-water quality at the household level rather than relying on service level of water supplies [38], [39]. Such field assessments of water quality will likely provide a more accurate assessment of the contribution that HWT makes in ensuring safe drinking-water.

More importantly, the lack of consistency and microbiological effectiveness raise important questions about the potential contribution of HWT in low- and middle-income countries in reducing the risk of waterborne diseases. As these results are largely consistent with those in similar case studies in Cambodia [29], India [24] and Zambia [28], it seems appropriate to reconsider the manner in which HWT is implemented in order to improve its consistency and effectiveness—conditions necessary to optimise its potential health impact.

## Supporting Information

S1 Figure **Distribution of Log_10_ reductions in TTC counts in paired source-drinking water samples among all samples (top) and among those pairs where the source water was CFU/100 mL>0.**
(TIF)

S1 Table Demographic characteristics of participating households in the urban and rural communities.
(DOCX)

S2 Table Characteristics of supplementation with untreated water among self-reported HWT users identified during the HWT practices survey.
(DOCX)

S3 Table Selected characteristics of HWT and water-drinking events during the home observations.
(DOCX)

S1 File Complete primary data file of baseline survey in rural setting.
(DTA)

S2 File Complete primary data file of baseline survey in urban setting.
(DTA)

S3 File Complete primary data file of HWT practices survey and spot check visits in rural setting.
(DTA)

S4 File Complete primary data file of HWT practices survey and spot check visits in urban setting.
(DTA)

S5 File Complete primary water quality data file of rural setting.
(DTA)

S6 File Complete primary water quality data file of urban setting.
(DTA)

S7 File Summary data of home observations (urban & rural settings).
(XLSX)

S1 Text Extra information gathered during IDI in both setting on time, taste and cost associated with boiling drinking water.
(DOCX)
