# Supplementary figures and images for: Assessing the Consistency and Microbiological Effectiveness of Household Water Treatment Practices by Urban and Rural Populations Claiming to Treat Their Water at Home: A Case Study in Peru
Source: PLoS One. 2014 Dec 18;9(12):e114997. doi: 10.1371/journal.pone.0114997 (PMC4270781; doi:10.1371/journal.pone.0114997)

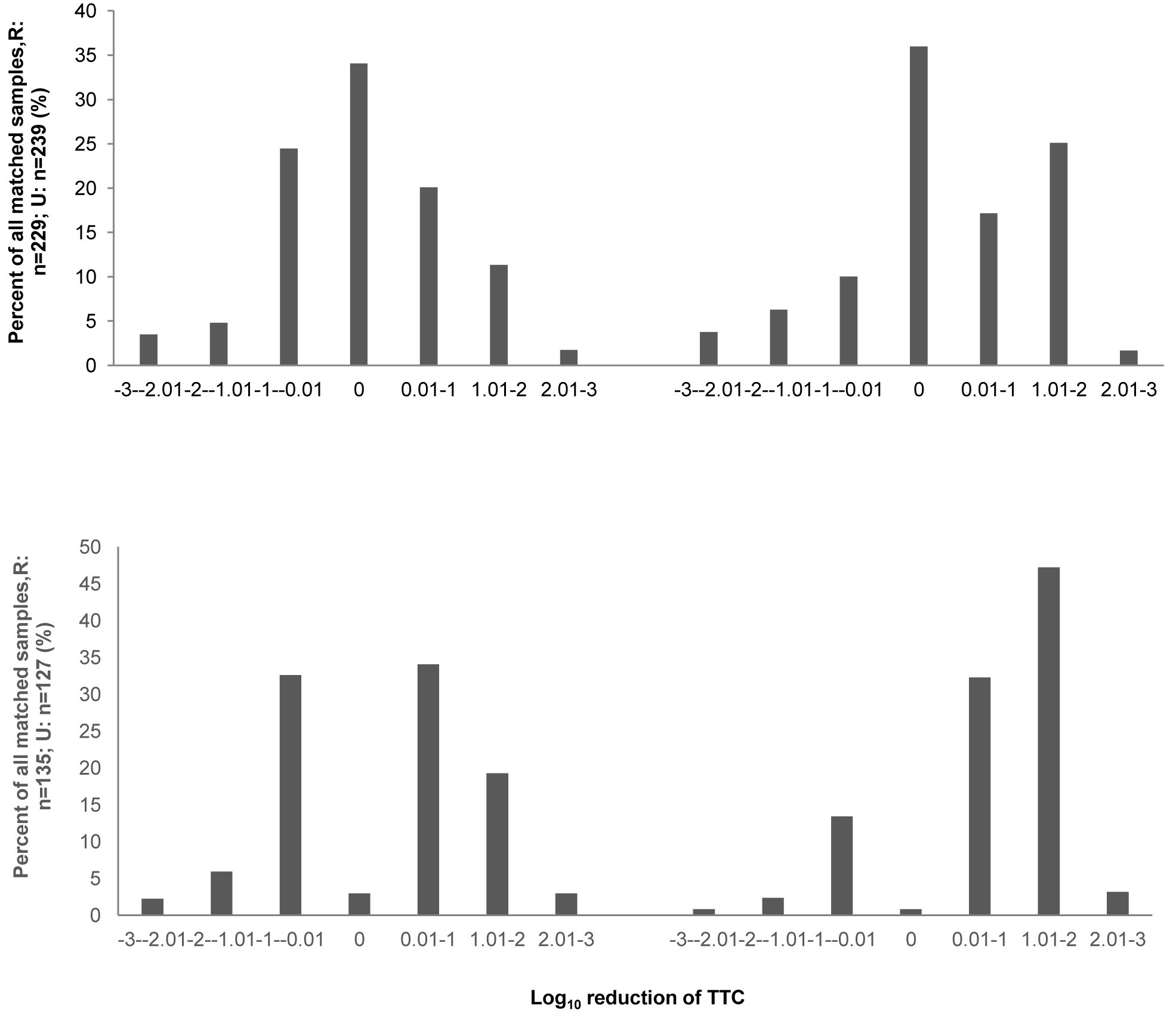

Supplement: S1 Figure — Distribution of Log10 reductions in TTC counts in paired source-drinking water samples among all samples (top) and among those pairs where the source water was CFU/100 mL>0. (TIF) [file pone.0114997.s001.tif]
