# Supplementary material for: Assessing the Consistency and Microbiological Effectiveness of Household Water Treatment Practices by Urban and Rural Populations Claiming to Treat Their Water at Home: A Case Study in Peru
Source: PLoS One. 2014 Dec 18;9(12):e114997. doi: 10.1371/journal.pone.0114997 (PMC4270781; doi:10.1371/journal.pone.0114997)
Supplement: S1 Table — Demographic characteristics of participating households in the urban and rural communities. (DOCX) [file pone.0114997.s002.docx]

**Table S1.** Demographic characteristics of participating households in the urban and rural communities.

| **Characteristic** | **Urban** | | **Rural** | |
| --- | --- | --- | --- | --- |
|  | **n** | **%** | **n** | **%** |
| Demographics |  |  |  |  |
| Total number of households | 209 | - | 230 | - |
| Total population | 807 | - | 813 | - |
| Number of children <5 years of age | 100 | 12.4 | 84.0 | 10.3 |
| Mean number of occupants per household (range) | 3.9 (1-13) |  | 3.5 (1 - 10) |  |
| Male headed households | 166 | 79.4 | 189 | 82.2 |
| Education of head of household |  |  |  |  |
| No education | 13 | 6.2 | 21 | 9.4 |
| Nursery | 1 | 0.5 | 1 | 0.4 |
| Primary | 92 | 44.0 | 163 | 72.8 |
| Secondary | 51 | 24.4 | 30 | 13.4 |
| Higher | 52 | 24.9 | 9 | 4.0 |
| Own |  |  |  |  |
| Electricity | 196 | 93.8 | 3 | 1.3 |
| Radio | 179 | 85.7 | 205 | 89.1 |
| TV | 182 | 87.1 | 11 | 4.8 |
| Mobile phone | 179 | 85.6 | 89 | 38.7 |
| Refrigerator | 42 | 20.1 | 0 | 0.0 |
| Mean number of rooms (range) | 4.5 (1-16) |  | 3.4 (1-10) |  |
| Mean number of sleeping rooms (range) | 1.8 (1-7) |  | 1.6 (1-6) |  |
| Source of drinking water |  |  |  |  |
| Piped water in dwelling/yard/plot | 202 | 96.6 | 196 | 85.6 |
| Standpipe | 0 | 0.0 | 1 | 0.4 |
| Spring water | 0 | 0.0 | 21 | 9.2 |
| Unprotected dug well | 4 | 1.9 | 5 | 2.2 |
| Bottled water | 1 | 0.5 | 0 | 0.0 |
| Other | 2 | 1.0 | 6 | 2.6 |
| Change water source in dry season | 4 | 1.9 | 3 | 1.3 |
| Reported HWT-use prior drinking | 162 | 77.5 | 163 | 70.9 |
| Method of HWT used^1^ |  |  |  |  |
| Boil | 162 | 77.5 | 160 | 69.6 |
| Use chlorine or bleach | 11 | 5.3 | 5 | 2.2 |
| Solar disinfection | 0 | 0.0 | 5 | 2.2 |
| Strain through a cloth | 1 | 0.5 | 1 | 0.4 |
| Let stand and settle | 2 | 1.0 | 1 | 0.4 |
| Have adequate sanitation facilities^2^ | 194 | 94.6 | 174 | 76.0 |
| Share toilet facilites^3^ | 38 | 19.1 | 7 | 3.2 |
| Main source of cooking fuel |  |  |  |  |
| Electricity | 1 | 0.5 | 0.0 | 0 |
| Gas | 122 | 58.4 | 4 | 1.7 |
| Firewood | 83 | 39.7 | 222 | 96.5 |
| Dung | 1 | 0.5 | 2 | 0.9 |
| Other | 2 | 1.0 | 0 | 0.0 |
| Do not cook at home | 0 | 0.0 | 2 | 0.9 |
| Cook in three-stone fire^4^ | 67 | 79.8 | 88 | 38.6 |
| ^1^Respondents may report multiple treatment methods, so the sum of treatment may exceed 100 percent | | | | |
| ^2^Includes flush/pour flush toilet to sewer or pit latrine, VIP latrines, pit latrines with slab and composting toilets | | | | |
| ^3^Only for those households that have access to a facility | | | | |
| ^4^Among solid fuel users | | | | |
