# Supplementary material for: Assessing the Consistency and Microbiological Effectiveness of Household Water Treatment Practices by Urban and Rural Populations Claiming to Treat Their Water at Home: A Case Study in Peru
Source: PLoS One. 2014 Dec 18;9(12):e114997. doi: 10.1371/journal.pone.0114997 (PMC4270781; doi:10.1371/journal.pone.0114997)
Supplement: S2 Table — Characteristics of supplementation with untreated water among self-reported HWT users identified during the HWT practices survey. (DOCX) [file pone.0114997.s003.docx]

**Table S2.** Characteristics of supplementation with untreated water among self-reported HWT users identified during the HWT practices survey.

| **Characteristic** | **Urban** | | **Rural** | |
| --- | --- | --- | --- | --- |
|  | **n** | **%** | **n** | **%** |
| Number of respondents | 81 |  | 68 |  |
| Respondent reports drinking untreated water in the home (supplements) | 28 | 34.6 | 46 | 67.7 |
| Reported frequency of consumption of untreated water in the home |  |  |  |  |
| Daily | 5 | 17.9 | 11 | 23.9 |
| 1-4 times per week | 11 | 39.3 | 18 | 39.1 |
| Regularly | 4 | 14.3 | 2 | 4.3 |
| Rarely | 8 | 28.6 | 14 | 30.4 |
| *Cross-checking reported data in supplementation* |  |  |  |  |
| Unavailability of treated water among self-reported non-supplementers^1^ | 3 | 5.9 | 4 | 21.1 |
| Number of households with children under 5 years | 29 |  | 20 |  |
| Participant reports that children under 5 years drink untreated water in the home | 8 | 27.6 | 11 | 55.0 |
| Reported frequency of consumption of untreated water in the home |  |  |  |  |
| Regularly/Weekly | 3 | 37.5 | 8 | 72.7 |
| Rarely | 4 | 50.0 | 3 | 18.2 |
| Don't know | 1 | 12.5 | 0 | 0.0 |
| Reported main reason for consumption of untreated water |  |  |  |  |
| Lack of boiled water at the time | 12 | 50.0 | 22 | 56.4 |
| Boiled water had not cooled | 3 | 12.5 | 1 | 2.6 |
| Dislike the taste of boiled water | 2 | 8.3 | 0 | 0.0 |
| Boiled water does not quench thirst | 2 | 8.3 | 3 | 7.7 |
| Desire of drinking raw water | 3 | 12.5 | 2 | 5.1 |
| Quicker | 2 | 8.3 | 4 | 10.3 |
| Other/No real reason | 0 | 0.0 | 7 | 17.9 |
| ^1^ Among households with water available at time of visit (U: n= 51, R: n=19) | | | | |
