# Supplementary material for: Assessing the Consistency and Microbiological Effectiveness of Household Water Treatment Practices by Urban and Rural Populations Claiming to Treat Their Water at Home: A Case Study in Peru
Source: PLoS One. 2014 Dec 18;9(12):e114997. doi: 10.1371/journal.pone.0114997 (PMC4270781; doi:10.1371/journal.pone.0114997)
Supplement: S3 Table — Selected characteristics of HWT and water-drinking events during the home observations. (DOCX) [file pone.0114997.s004.docx]

**Table S3:** Selected characteristics of HWT and water-drinking events during the home observations.

| **Characteristic** | **Urban** | **Rural** |
| --- | --- | --- |
| Number of households | 12 | 15 |
| Number of days under observation | 3 | 3 |
| Mean hours of observation per household | 12.8 | 14.1 |
| Time of observation | 08:00 - 13:30 | 06:30- 11:30 |
| Total number of children <5 yr (mean) | 15 (1.3) | 22 (1.5) |
| **HWT-related events** |  |  |
| Total HWT events observed | 8 | 16 |
| Number of households where no HWT events observed (%) | 5 (41.7%) | 7 (46.7%) |
| Number of households reporting daily HWT-use (%)^1^ | 9 (81.2%) | 6 (46.2) |
| Number of households reporting daily use and ≥1 HWT event observed (%) | 5 (55.5%) | 3 (50.0%) |
| Total number of HWT events observed among households reporting daily use (mean)^1^ | 6 (0.7) | 6 (1.0) |
| **Drinking water-related events** |  |  |
| Total drinking events observed | 127 | 128 |
| Total number of water drinking events observed (mean): Adults | 35 (2.9) | 40 (2.7) |
| Total number of water drinking events observed (mean): Children | 31 (2.6) | 42 (2.8) |
| Number of households where no water drinking events observed | 1 | 2 |
| Participant reported not consuming untreated water (non-supplementation) | 3 | 4 |
| Participant reported children <5s not consuming untreated water^2^ | 6 | 2 |
| Mean percentage of untreated water from all water consumed^3^: Adults | 63.3 | 57.6 |
| Mean percentage of untreated water from all water consumed^3:^ Children | 48.1 | 64.8 |
| ^1^Urban: One household did not complete IDI. Rural: One household did not complete IDI & another reported not treating | |  |
| ^2^Two missing values |  |  |
| ^3^Drinking water classified as treated, untreated or unknown if HWT status was not determined (Unknown: U: 42.4, R: 2.6) | |  |
