# Supplementary material for: Assessing the Consistency and Microbiological Effectiveness of Household Water Treatment Practices by Urban and Rural Populations Claiming to Treat Their Water at Home: A Case Study in Peru
Source: PLoS One. 2014 Dec 18;9(12):e114997. doi: 10.1371/journal.pone.0114997 (PMC4270781; doi:10.1371/journal.pone.0114997)
Supplement: S1 Text — Extra information gathered during IDI in both setting on time, taste and cost associated with boiling drinking water. (DOCX) [file pone.0114997.s012.docx]

**Text S1**

Reported boiling practices during the in-depth interviews

*Time*

Respondents reported that boiling would require 5-30 minutes- depending on i. fuel type, ii. state of the fire, iii. volume of water, iv. type of pot, and v. time available—the more busy the respondent was, the shorter the water would be boiled for. None of the households reported that boiling would be a time consuming task, and all believed it was worthwhile.

*Taste*

Most respondents in both settings reported that boiling would change the taste of the water (80% urban, 100% rural). In the urban context all agreed that the taste of the boiled water was not as pleasant or satisfying while in the rural context, 40% thought so.

*Cost*

In both settings, respondents seemed not to attach an economical cost to boiling (U: %, R: 100%) as firewood would be collected and not purchased. Only one urban respondent reported that when firewood was not available and gas would be used instead, then boiling would cost but the participant was unable to assess how much fuel would be used specifically for boiling as opposed to other activities.
